# The Dynamics of Long Terminal Repeat Retrotransposon Proliferation and Decay Drive the Evolution of Genome Size Variation in Capsicum

**DOI:** 10.3390/plants14142136

**Published:** 2025-07-10

**Authors:** Qian Liu, Pinbo Liu, Shenghui Wang, Jian Yang, Liangying Dai, Jingyuan Zheng, Yunsheng Wang

**Affiliations:** 1 College of Plant Protection, Hunan Agricultural University, Changsha 410128, China; qianliu7427@163.com (Q.L.); liupinbo1999@163.com (P.L.); wangshenghui1029@163.com (S.W.); v3473119071@163.com (J.Y.); daily@hunau.net (L.D.); 2 Hunan Vegetable Research Institute, Hunan Academy of Agricultural Sciences, Changsha 410125, China

**Keywords:** *Capsicum*, comparative genomics, genome size evolution, transposable element, long terminal repeat retrotransposon

## Abstract

*Capsicum* (pepper) is an economically vital genus in the *Solanaceae* family, with most species possessing about 3 Gb genomes. However, the recently sequenced *Capsicum rhomboideum* (~1.7 Gb) represents the first reported case of an extremely compact genome in *Capsicum*, providing a unique and ideal model for studying genome size evolution. To elucidate the mechanisms driving this variation, we performed comparative genomic analyses between the compact *Capsicum rhomboideum* and the reference *Capsicum annuum* cv. CM334 (~2.9 Gb). Although their genome size differences initially suggested whole-genome duplication (WGD) as a potential driver, both species shared two ancient WGD events with identical timing, predating their divergence and thus ruling out WGD as a direct contributor to their size difference. Instead, transposable elements (TEs), particularly long terminal repeat retrotransposons (LTR-RTs), emerged as the dominant force shaping genome size variation. Genome size strongly correlated with LTR-RT abundance, and multiple LTR-RT burst events aligned with major phases of genome expansion. Notably, the integrity and transcriptional activity of LTR-RTs decline over evolutionary time; older insertions exhibit greater structural degradation and reduced activity, reflecting their dynamic nature. This study systematically delineated the evolutionary trajectory of LTR-RTs—from insertion and proliferation to decay–uncovering their pivotal role in driving *Capsicum* genome size evolution. Our findings advance the understanding of plant genome dynamics and provide a framework for studying genome size variation across diverse plant lineages.

## 1. Introduction

Genome size is a fundamental aspect of biodiversity that remains relatively constant within species, while exhibiting remarkable variation across different organisms [1,2,3,4]. Among eukaryotes, genome size variation exceeds 640,000-fold based on data from over 15,000 species [5], with land plants showing particularly striking differences of approximately 2400-fold [6]. Plant genome sequences can be classified into repetitive and non-repetitive components, with repetitive sequences comprising the majority of most plant genomes.

Transposable elements (TEs) represent the most abundant category of repetitive sequences in plant genomes [7,8]. Numerous studies have demonstrated that TEs play a crucial role in angiosperm genome size variation [9,10,11,12,13]. Among the various factors affecting genome size changes, the differential accumulation of TEs, particularly long terminal repeat retrotransposons (LTR-RTs), is the primary driver of genome variation, alongside polyploidization resulting from whole-genome duplication (WGD) events [14,15,16,17,18,19]. LTR-RTs self-replicate through copy–transposition mechanisms and accumulate over time, progressively increasing genome size [20,21]. For instance, in the small genome of *Arabidopsis thaliana* (~157 Mb), LTR-RTs constitute only about 5.6% [22], while in *Oryza sativa* (~430 Mb), they represent 15–25% of the genome [23,24], and in *Zea mays* (~2 Gb), they account for over 74.6% of the genome [25]. Understanding how LTR-RTs influence genome size is therefore essential for investigating genome size evolution.

The genus *Capsicum*, an important member of the *Solanaceae* family, serves as an ideal model for studying LTR-RT dynamics and distribution, due to its relatively large genomes and significant range in genome size (from 1.7 Gb to 3 Gb). With over 70% of these genomes composed of repetitive sequences [26,27], *Capsicum* offers a unique opportunity to investigate the mechanisms of genome expansion and contraction. Despite its agricultural and economic significance, comprehensive understanding of the driving mechanisms behind genome size variation in *Capsicum* remains limited, particularly regarding the timing and composition of TE bursts.

In this study, we conducted a comparative analysis of two *Capsicum* genomes with substantial size differences: *Capsicum rhomboideum* (CrT2T, 1.7 Gb) [28] and *Capsicum annuum* cv. CM334 (CM334, 2.9 Gb) [26]. CrT2T represents the first reported case of an extremely compact genome in *Capsicum*, serving as an ideal model for investigating genome size evolution. CM334, although not assembled to a telomere-to-telomere (T2T) level, is the most extensively annotated and widely cited reference genome of *Capsicum*. Our preliminary analysis of other large *Capsicum* genomes (e.g., the 3 Gb Ca59) revealed that the repeat composition and insertion time distribution are highly consistent with those of CM334. Based on their structural representativeness and extensive application in previous studies, CrT2T and CM334 were selected for comparison. This comparison provided an excellent opportunity to investigate how recent LTR-RTs bursts have influenced genome size variation within closely related species. By quantitatively assessing TE composition, abundance, and evolutionary dynamics, our results further confirm the critical role of TEs, especially LTR-RTs, in genome size evolution, particularly through episodic LTR-RT bursts that cause significant genome size variation over relatively short evolutionary periods.

## 2. Materials and Methods

### 2.1. Transposable Element Annotation and Classification

We constructed species-specific repeat libraries for each *Capsicum* genome by integrating repeat sequences from the Repbase database [29], Dfam database [30], and de novo repeats generated by RepeatModeler (v2.1) (https://github.com/Dfam-consortium/RepeatModeler accessed on 23 April 2025). Then, based on the integrated repeat sequence database, the repetitive elements in each genome were annotated and masked using RepeatMasker (v4.1.5) [31] with the following parameters: -e rmblast -xsmall -s -gff –cutoff 255 –frag 60000 -no_id. To accurately identify full-length LTR-RTs in the genomes, we first performed de novo annotation using LTR_Finder (v1.2) [32] and LTRharvest (v1.6.5) [33] to detect candidate LTR-RT elements. These candidate LTR-RTs were then filtered and integrated using LTR_retriever (v2.9.4) [34] to generate a high-quality, full-length LTR-RT sequence dataset. We identified 10,184 full-length LTR-RTs in CrT2T and 4073 in CM334, respectively.

TEsorter (v1.4.6) [35] was subsequently employed to further classify and analyze the annotated TEs and LTR-RTs, based on HMM profiles from the REXdb-Viridiplantae database [36], using the following parameters: –max-evalue 0.001 –min-coverage 20 –min-probability 0.5. This classification was primarily based on the presence and sequential order of five conserved protein domains: capsid protein (GAG), aspartic proteinase (AP), integrase (INT), reverse transcriptase (RT), and RNase H (RH). After re-annotation, the number of intact LTR-RTs decreased to 3681 in CM334 and 9831 in CrT2T. Within the Ty1 retrotransposon superfamily, elements were classified into several clades, including Ale, Alesia, Angela, Bianca, Ikeros, Ivana, SIRE, TAR, and Tork. Meanwhile, the Ty3 retrotransposon was further divided into Athila, CRM, Galadriel, Ogre, Reina, and Tekay clades.

### 2.2. Comparative Genomic Analysis

We used JCVI (v1.4.2) [37] software to identify syntenic blocks between the two genomes and generate collinearity plots. To understand the timing and frequency of whole-genome duplication events in peppers, we calculated synonymous substitution rates (Ks values) for syntenic gene pairs using WGDI (v0.6.5) [38] and generated Ks frequency distribution plots. To remove the influence of tandem duplications on WGD event time estimates, we set the parameters in the collinearity block as follows: “multiple = 2; evalue = 1 $\times 10^{-5}$; score = 100; grading = 50, 40, 25; mg = 40, 40; pvalue = 1; repeat_number = 3”. We then calculated the timing of whole-genome duplication events using the molecular clock formula T = Ks/2r [39]. We employed an average substitution rate (r) of 7 × 10^−9^ substitutions per site per year, which was based on estimates from *Arabidopsis thaliana* [40], to estimate the timing of WGD events. This rate has also been widely adopted in previous studies on *Capsicum* genomes [28].

### 2.3. Phylogenetic Analysis

Nonredundant protein sequences from 24 species were prepared for ortholog analyses (Supplementary Table S1). Orthologs and orthogroups were then inferred using OrthoFinder (v2.5.5) [41] with the default settings and ’-M msa’ activation. The longest predicted protein of each individual gene was used as the representative input for the OrthoFinder analysis. IQ-TREE (v2.2.5) [42] was used to construct maximum likelihood phylogenetic trees with the following parameters: -B 1000 -alrt 1000 -nt 20, with rice (*Oryza sativa*) as an outgroup. TimeTree (http://www.timetree.org/ accessed on 23 April 2025) is a public evolutionary time database and tool designed for querying and comparing species divergence times. It integrates a vast amount of divergence time estimates from various publications, along with its own estimations. In this study, divergence time estimates were obtained from TimeTree, and outliers were excluded to determine the lower and upper bounds of uniform calibration priors. Based on these refined estimates, the calibration values were set as 0.002–0.145, 0.017–0.021, and 1.114–1.239 for the most recent common ancestor of *Nicotiana*, *Solanum*, and *Capsicum*, respectively. The MCMCTree program in PAML (v4.10.7) [43] was used to analyze amino acid substitution models and estimate divergence times. Subsequently, CAFE5 [44] was employed to infer gene gain and loss rates across genomes. Orthogroups identified by OrthoFinder were treated as distinct gene families and used as input for CAFE5 analysis.

### 2.4. TE Dynamics Analysis

The insertion times of the intact LTR-RTs were calculated using LTR_retriever based on the formula T = Ks/2r [39], where K represents the sequence divergence(substitutions per site) between the two LTR-RTs of a single retrotransposon, and r is the nucleotide substitution rate. An average substitution rate (r) of 7 × 10^−9^ was applied to estimate the insertion times of LTR-RTs. The script calcDivergenceFromAlign.pl from the RepeatMasker software was utilized to calculate the Kimura substitution level of TEs, providing insights into their sequence divergence. The resulting data were then processed and analyzed using R, where statistical methods were applied to summarize the distribution of substitution levels. Additionally, visualization techniques in R were employed to generate informative plots, facilitating the interpretation of TE divergence patterns.

### 2.5. Transcriptional Activity Analysis

RNA sequence data were downloaded from the NCBI Sequence Read Archive (SRA) using prefetch (v2.11.2) from the SRA Toolkit (https://github.com/ncbi/sra-tools accessed on 20 May 2025). The downloaded SRA files were converted to FASTQ format using fastq-dump (v2.11.2) (https://github.com/ncbi/sra-tools accessed on 20 May 2025) with the –split-3 and –gzip options to generate compressed paired-end reads. Quality control was performed using FastQC (v0.12.1) [45] to assess sequence quality, and MultiQC (v1.23) [46] was used to aggregate and visualize quality metrics. Read trimming and filtering were performed using Trimmomatic (v0.39) [47] with the ILLUMINACLIP option. The parameters used were LEADING:3, TRAILING:3, SLIDINGWINDOW:4:15, and MINLEN:36, ensuring high-quality reads for downstream analysis. The STAR (2.7.11b) [48] aligner was used to generate the genome index, with the –sjdbOverhang parameter set to 99, optimizing the index for the 100 bp paired-end reads. Read alignment was performed using STAR (2.7.11b). The output BAM file, along with the reference genome annotation file and the TEsorter (v1.4.6) structural classification file, were provided as input for TEcount (v2.2.3) [49] to perform transcript quantification. Counts for mapped reads were normalized by transcripts per million (TPM). To ensure the reliability of the detected transposable element expression, only LTR-RTs elements with conserved RT domain regions and TPM > 1 were considered active and retained for downstream analysis.

## 3. Results

### 3.1. Comparative Genomics and Evolution of *Capsicum* Genomes with Divergent Sizes

To explore the evolutionary history and potential causes of the variation in genome size in *Capsicum*, we performed comparative genomic and phylogenetic analysis using *C. rhomboideum* (CrT2T, 1.7 Gb), *C. annuum* cv. CM334 (CM334, 2.9 Gb), and 22 additional angiosperm genomes. These included four *Capsicum* genomes of approximately 3 Gb, one *Physalis* genome, six *Solanum* genomes, and four *Nicotiana* genomes (Table S1).

**Figure 1:**
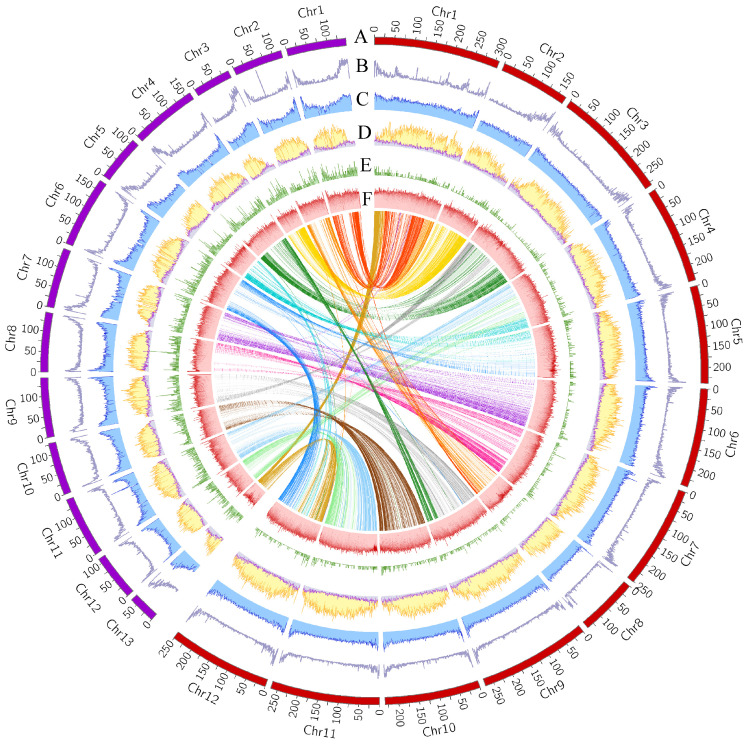
Global view of the two pepper genomes. Track A presents the pseudochromosomes of peppers (Red: *C. annuum* cv. CM334, Violet purple: *C. rhomboideum*). Track B shows the density of genes(Purple). Track C denotes the distribution of TE(Blue). Track D shows the density distribution of the Ty3 retrotransposon (Yellow) and the Ty1 retrotransposon (Light purple). Track E represents the distribution of LTR-RTs with transcriptional activity(Light green). Track F represents the Kimura values of TEs (Light pink).

Statistical analysis revealed that despite the CrT2T genome being only about half the size of the CM334 genome, both genomes contain similar numbers and total lengths of protein-coding genes, with comparable gene distribution patterns showing higher gene density at chromosome ends (Figure 1 Track B). CrT2T contains 33,512 genes with a total length of 181,798,887 bp, while CM334 has 31,600 genes totaling 142,495,079 bp. In particular, despite its smaller genome, CrT2T has one more chromosome than CM334, an unusual characteristic that distinguishes it from other species of *Capsicum* (Table 1). These results indicate that protein-coding gene number, length, and chromosome count do not correlate with genome size, as CrT2T exceeds CM334 in all three aspects despite its smaller genome.

**Figure 2:**
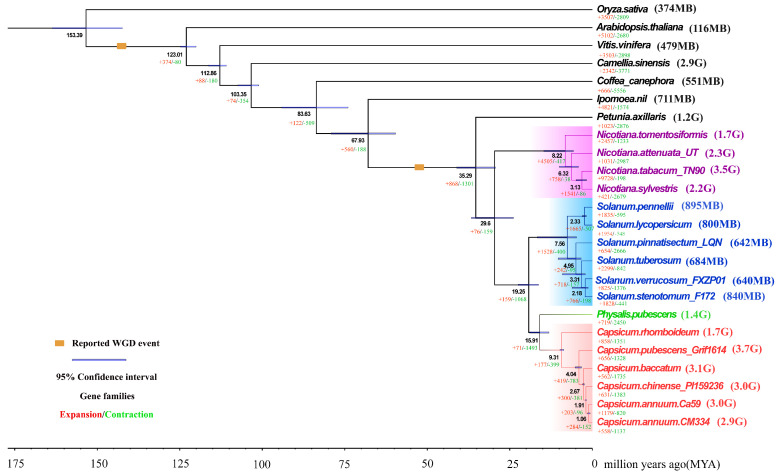
Phylogenomic analysis of *Capsicum* and related angiosperm species. Whole genome duplication (WGD) events, and gene family expansion/contraction statistics are marked on the MCMC phylogenetic tree, which was constructed using single-copy orthologs. Clade colors represent taxonomic groups: Capsicum (red), Solanum (blue), Nicotiana (purple), Physalis (green), and distantly related species (black).

Self-collinearity dot plots of the two genomes revealed a large number of homologous sequences (Figure S1). WGD analysis identified two distinct peaks at Ks = 0.75 and Ks = 2.0 in the Ks value distributions of both genomes (Figure S2). Using the molecular clock formula T = Ks/2r [39], we estimated that both CrT2T and CM334 genomes underwent WGD events approximately 53 MYA and 143 MYA (Figure 2). Phylogenetic analysis indicated that among all analyzed species, *Capsicum* is most closely related to *Physalis*, with a divergence time of approximately 15.71 MYA. In addition, CrT2T exhibited a more distant phylogenetic relationship from other *Capsicum* species, representing the earliest divergence within the genus, around 9.31 million years ago (Figure 2). This divergence pattern is consistent with previous studies [28,50,51]. The genome size difference between CrT2T and CM334 gradually emerged after this divergence event, which occurred significantly later than the WGD events. Collinearity analysis revealed that despite the markedly smaller genome size of CrT2T and its more distant phylogenetic relationship to CM334, the two genomes still exhibit strong gene-level and chromosomal collinearity (Figure 1, Figures S3 and S4). This indicates that the additional chromosome in CrT2T likely originated from chromosomal fission and rearrangement events.

In summary, these results demonstrate that CrT2T and CM334 have undergone complex evolutionary processes, leading to substantial differences in genome size and chromosome number, while gene number differences remain relatively limited. Both whole-genome duplication (WGD) events occurred before species divergence and therefore were not the direct cause of the genome size differences. Instead, after the divergence of CrT2T from the *Capsicum* genus 9.31 MYA, CM334’s genome likely expanded due to specific factors causing sequence proliferation, while CrT2T appeared unaffected, maintaining a smaller genome size.

### 3.2. Transposable Element Composition and Abundance Correlate with Genome Size

We identified transposable elements in CM334 and CrT2T genomes using a combination of homology-based searches and ab initio modeling. RepeatMasker identified that repetitive sequences comprise 85.84% (~2.49 Gb) of the CM334 genome and 83.24% (~1.42 Gb) of the CrT2T genome. In the two *Capsicum* genomes, the total amount of repetitive sequences increases with genome size. Specifically, transposable elements (TEs) constitute 85.15% (~2.47 Gb) of the CM334 genome and 82.38% (~1.41 Gb) of the CrT2T genome. The genomic distribution of TEs positively correlates with that of genes, with both primarily concentrated at chromosome ends (Figure 1 Tracks B–C).

**Figure 3:**
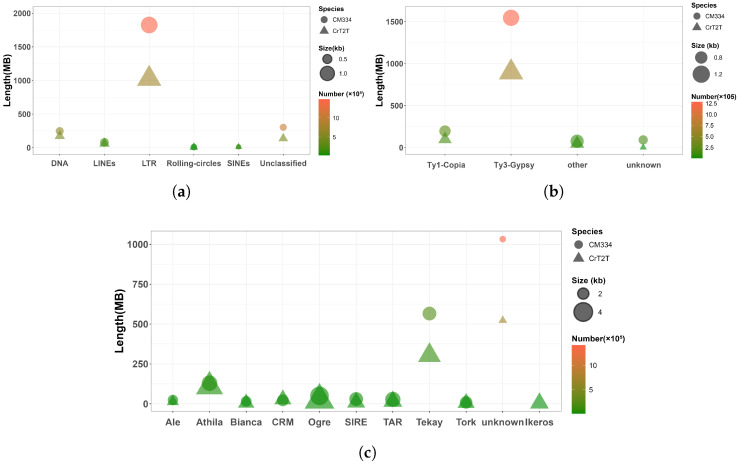
Composition and length of repetitive sequence elements in pepper genomes. (**a**) Total length of various repetitive sequence elements; (**b**) total length of Ty3 retrotransposon and Ty1 retrotransposon superfamilies within LTR-RT elements; (**c**) total length of different families within LTR-RT elements.

Further comparison of Class I and Class II transposons revealed that Class I elements show more significant differences between the two genomes, with approximately 1.79 Gb in CM334 but only 1.02 Gb in CrT2T. LTR-RT elements are particularly divergent, comprising 1.70 Gb in CM334 but only 0.95 Gb in CrT2T (Figure 3b). Using TEsorter (v1.4.6) to reclassify LTR-RT superfamilies and families, we found that the Ty3 Retrotransposon superfamily is significantly more abundant than the Ty1 retrotransposon superfamily in both genomes (Figure 3b). Additionally, the Ty3 retrotransposon superfamily, especially the Tekay family, is notably more abundant in the CM334 genome compared to CrT2T, while other families show similar abundances (Figure 3c). Ty1 retrotransposon and Ty3 retrotransposon exhibit distinctly different distribution patterns across chromosomes, with the Ty1 retrotransposon primarily concentrated at chromosome ends similarly to genes, while Ty3 retrotransposon is predominantly distributed in central chromosome regions (Figure 1 Track D).

In conclusion, the significant difference in the size between the two pepper genomes is mainly driven by the expansion of repetitive sequences, with LTR-RT elements playing a key role. In particular, the extensive proliferation of the Tekay family within the Ty3 retrotransposon superfamily in the CM334 genome compared to CrT2T probably represents a major contributor to their genome size difference.

### 3.3. Transposable Element Insertion Patterns Reflect Dynamic Changes in Genome Size

To further investigate the characteristics of LTR-RT composition in pepper genomes, we identified intact LTR-RTs in both genomes. Using LTRharvest, LTR_FINDER_parallel, and LTRharvest, we identified 4073 intact LTR-RTs totaling 27,759,903 bp in CM334 and 10,184 intact LTR-RTs totaling 91,362,481 bp in CrT2T. After reclassification with TEsorter, the number of intact LTR-RTs decreased to 3681 in CM334 and 9830 in CrT2T.

**Figure 4:**
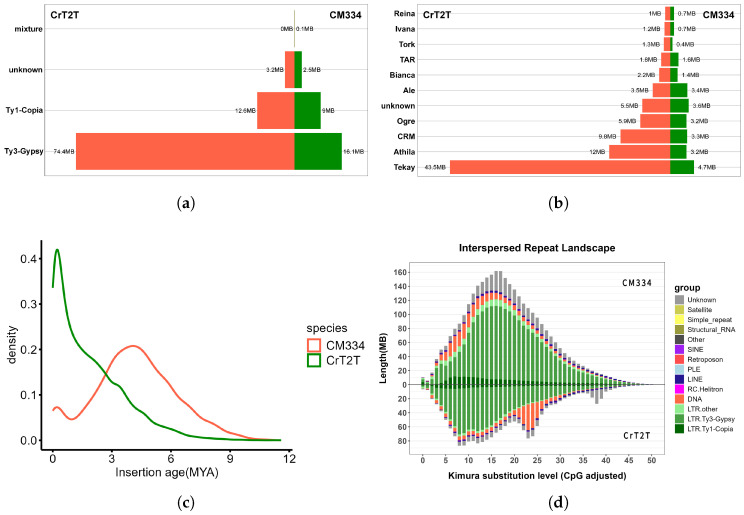
Comparison of intact LTR-RT elements and insertion dynamics. (**a**) Proportion of Ty3 retrotransposon and Ty1 retrotransposon superfamilies within intact LTR-RT elements; (**b**) proportion of different families within LTR-RT elements; (**c**) intact LTR-RT insertion age distribution; (**d**) Kimura substitution analysis of TE landscape: shows the Kimura substitution analysis of TEs in the CM334 and CrT2T genomes.

In the CM334 genome, these included 1681 from the Ty1 retrotransposon superfamily and 1981 from the Ty3 retrotransposon superfamily, while in the CrT2T genome, there were 2235 from the Ty1 retrotransposon and 7534 from the Ty3 retrotransposon. Consistent with whole-genome LTR-RT classification, the number of Ty3 retrotransposon superfamily elements exceeded Ty1 retrotransposon in intact LTR-RTs. However, unlike the whole-genome analysis, CM334 contained fewer elements of each type than the CrT2T genome, particularly Ty3 retrotransposon superfamily elements, while Ty1 retrotransposon showed relatively smaller differences (Figure 4a). In the family classification of intact LTR-RTs, the proportion of the Tekay family in CrT2T significantly exceeded that in CM334 (Figure 4b), possibly related to recent LTR-RT bursts. The number and total length of intact LTR-RTs exhibited a negative correlation with genome size, with approximately 2.5 times more intact LTR-RTs in the smaller CrT2T genome than in CM334.

To investigate LTR-RT expansion events in pepper genomes, we estimated the insertion times of all intact LTR-RTs based on sequence divergence. The results showed that the CM334 genome exhibited two bursts of LTR-RT insertions, occurring approximately 0.1 million years ago (Mya) and 3.9 Mya, while CrT2T showed only one major LTR-RT expansion event around 0.1 Mya (Figure 4c). LTR-RT expansion frequency positively correlates with genome size. To further verify the dynamic changes in LTR-RT insertion times, we calculated Kimura values for all TE sequences. Compared to CrT2T, CM334 showed generally higher Kimura values, with peaks around 15, while CrT2T peaks were around 10 (Figure 4d), consistent with the trend observed in intact LTR-RT burst times.

Although CrT2T experienced fewer bursts, its later burst timing resulted in a greater number of preserved intact LTR-RTs. In contrast, CM334 underwent two LTR-RT burst events, with the 0.1 Mya burst being relatively small and the 3.9 Mya burst occurring earlier. Over time, many originally intact LTR-RTs were gradually disrupted by subsequently inserted sequences and lost their integrity, resulting in fewer detected intact LTR-RTs. This explains why CrT2T, despite having a smaller genome than CM334, contains significantly more intact LTR-RTs.

### 3.4. Transposable Element Transcriptional Activity Identification

To compare the transcriptional activity and distribution patterns of transposable elements (TEs) in the CM334 and CrT2T pepper genomes, we mapped RNA-seq data to TE-annotated regions and intact LTR-annotated regions, focusing on the expression of RT regions to identify TEs with transcriptional activity in both genomes.

In the CM334 genome, we identified 1271 TEs with RT domain expression, including 687 Ty1 retrotransposons and 358 Ty3 retrotransposons, among which 97 were intact LTR-RT elements. To distinguish autonomous TE expression from possible passive transcription driven by nearby genes, we defined “co-transcribed” TEs as those whose annotated coordinates overlap with gene features (including exons, UTRs, and introns) or lie within 2 kb upstream or downstream of annotated genes. Based on this criterion, 509 of the expressed elements in CM334 are likely the result of passive co-transcription with host genes rather than autonomous transposon activity. These include 300 Ty1 and 112 Ty3 elements. In the CrT2T genome, 1800 TEs with RT domain expression were detected, including 981 Ty1 retrotransposons and 730 Ty3 retrotransposons, with 298 intact LTR-RT elements. Using the same definition, 737 elements were identified as likely passively co-transcribed with host genes, comprising 536 Ty1 and 172 Ty3 elements (Table 2). After excluding those potentially affected by host gene co-transcription, the number of transcriptionally active Ty3 elements in CrT2T was significantly higher than in CM334, while the number of Ty1 elements remained largely comparable between the two genomes.

**Figure 5:**
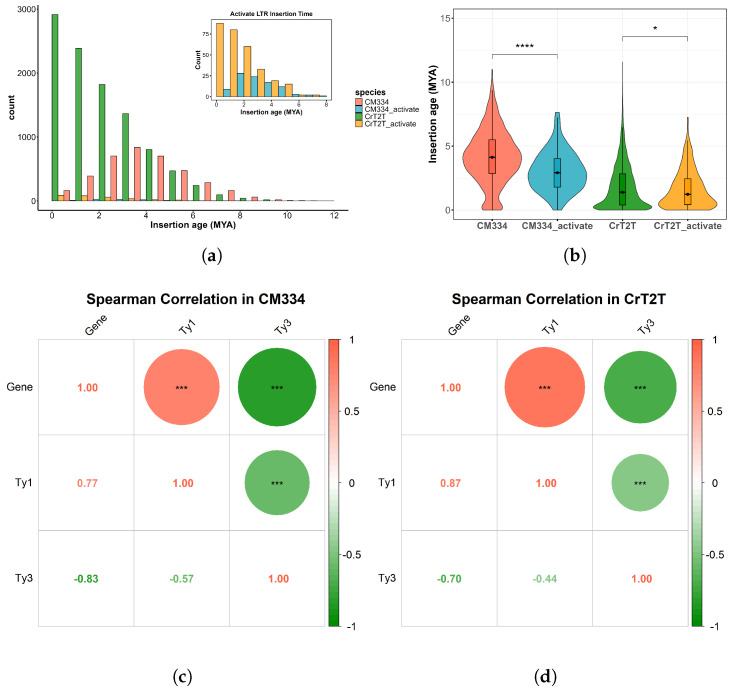
Comparison of insertion ages of intact LTR-RTs and transcriptionally active LTR-RTs. (**a**) Distribution of insertion ages of intact LTR-RTs in the CM334 and CrT2T genomes, with an inset showing the distribution of insertion ages of transcriptionally active LTR-RTs. (**b**) Significant differences in the insertion age distribution between intact LTR-RTs and transcriptionally active LTR-RTs in the CM334 and CrT2T genomes.Asterisks indicate statistical significance levels: * p<0.05, *** p<0.001, **** p<0.0001. (**c**) Spearman correlation between gene and LTR-RT densities in CM334. Heatmaps were generated based on non-overlapping sliding windows, showing the spatial correlation across the genome. Asterisks denote statistically significant correlations (* p<0.05, *** p<0.001). (**d**) Spearman correlation between gene and LTR-RT densities in CrT2T. Heatmaps were generated based on non-overlapping sliding windows, showing the spatial correlation across the genome. Asterisks denote statistically significant correlations (* p<0.05, *** p<0.001).

Statistical analysis of insertion times for transcriptionally active intact LTR-RTs revealed that in the CM334 genome, LTR-RTs with transcriptional activity were primarily inserted around 2 Mya, later than the major LTR-RT expansion in this genome. In the CrT2T genome, transcriptionally active LTR-RTs mainly originated from the LTR-RT burst event at 0.1 Mya (Figure 5a). Furthermore, the distribution of insertion times between all intact LTR-RTs and those with transcriptional activity showed significant differences, particularly evident in the CM334 genome (Figure 5b). TEs in CM334 mostly originated earlier, gradually accumulating mutations over time and losing structural integrity and functional activity, while the TEs in CrT2T arose more recently, thus maintaining higher structural integrity and functional activity. This difference also explains why significantly more intact LTR-RTs were detected in the CrT2T genome compared to the CM334 genome.

Notably, although Ty3 retrotransposon elements constitute approximately 53% and Ty1 retrotransposon elements only about 6% in both genomes, transcriptionally active RT components are predominantly Ty1 retrotransposon, accounting for over 50% of the total (Table 2). Additionally, examination of the Ty1 retrotransposon superfamily distribution revealed that Ty1 retrotransposon elements are primarily located in gene-rich regions (Figure 1 Track B, Figure 1 Track D). Correlation heatmaps of element distribution across the two genomes further supported this observation: in both genomes, Ty1 retrotransposons are primarily distributed in gene-rich regions, whereas Ty3 retrotransposons are mainly located in the gene-poor areas (Figure 5c,d). This characteristic suggests that the Ty1 retrotransposon superfamily may play a role in regulating specific genes in the genome, maintaining high stability and transcriptional activity throughout evolution. In contrast, Ty3 retrotransposon elements show a relatively unstable structure and function, being more prone to losing transposon activity. The number of Ty3 retrotransposon elements with transcriptionally active RT expression domains differs significantly between the two genomes, with considerably more in the CrT2T genome than in the CM334 genome, while differences in the Ty1 retrotransposon superfamily are relatively minor. Given the high proportion of Ty3 retrotransposon superfamily elements in the genome, we hypothesize that Ty3 retrotransposon element transcriptional activity may be closely related to dynamic changes in genome size.

## 4. Discussion

In eukaryotes, genome size evolution is typically unrelated to chromosome number or phylogenetic relationships, but primarily results from genome expansion and gene loss or deletion [4]. The main mechanisms of genome expansion include genome polyploidization and transposable element accumulation [12,52,53,54,55,56]. In this study, we compared two *Capsicum* species with significantly different genome sizes to investigate the key factors responsible for these substantial genome size differences.

Firstly, by estimating the timing of whole genome duplication (WGD) events, we found that two ancient WGD events occurred in the common ancestor of *Capsicum*, predating the divergence of the two species studied. This excludes the possibility that WGD events caused the observed chromosome size differences. Secondly, synteny analysis revealed that despite the significant differences in genome size and chromosome number between CrT2T and CM334, the two genomes still maintain strong gene-level and chromosomal synteny (Figure 1,  Figures S3 and S4). This suggests that the difference in chromosome number is primarily due to chromosomal rearrangement events rather than large-scale gene or sequence loss or amplification, thereby ruling out major chromosomal restructuring as a direct cause of genome size variation. This finding is consistent with previous studies, which showed that the 13 chromosomes of CrT2T originated from a 12-chromosome ancestral genome through multiple chromosomal rearrangement events [51]. Finally, through TE annotation in the two pepper genomes, we discovered significant differences in TE content, particularly in the Ty3 retrotransposon superfamily of LTR-RT elements. This indicates a strong correlation between genome size evolution trends and Ty3 retrotransposon elements. Previous comparative studies in rice (*Oryza*) [57], sunflower (*Helianthus*) [58], *Solanum* [59], and grape (*Vitis vinifera*) [60] have demonstrated strong correlations between retrotransposon and genome size evolution. Genome size changes are primarily driven by rapid expansion of retrotransposon families, sometimes accompanied by extensive structural rearrangements in certain subclasses. These studies suggest that estimating LTR-TE insertion times can help infer evolutionary trends.

Combining *Capsicum* species divergence time (Figure 2), LTR-RT and TE insertion times (Figure 4c,d), and LTR-RT transcriptional activity identification results, we hypothesize that around 9.31 MYA, influenced by environmental or specific factors, CM334 genome sequences rapidly proliferated, leading to genome expansion. However, CrT2T appeared unaffected by this event and maintained a smaller genome size. During this process, CrT2T gradually diverged from other *Capsicum* species, forming significant genome size differences.

Genome size variation in CM334 occurred earlier than in CrT2T, with most TEs originating from ancient expansion events. In contrast, CrT2T exhibited stronger dynamic activity in recent times, with its TEs primarily derived from recent proliferation. Owing to the intrinsic structural instability and rapid evolutionary dynamics of LTR-RTs in plant genomes [20,34], they are more vulnerable to mutation accumulation—such as deletions, truncations, and rearrangements—compared to genes. Most LTR-RTs rapidly lose their activity after insertion, due to structural degeneration and epigenetic silencing. Consequently, CrT2T retains more structurally intact and transcriptionally active LTR-RTs than CM334, where the majority of LTR-RTs were inserted earlier. This also explains the generally low proportion of RT domain expression among intact LTR-RTs (2.66% in CM334 and 3.04% in CrT2T).

Previous research has demonstrated that the Ty3 retrotransposon mediated genome size evolution in *Brassicaceae* plants [61]. In our study, we similarly found that the Ty3 retrotransposon comprises an extremely high proportion of pepper genomes and the number of transcriptionally active Ty3 elements detected in the CrT2T genome was significantly higher than that in CM334. Based on the LTR-RT insertion time analysis, the CrT2T genome shows greater recent instability in genome size, whereas the CM334 genome appears to have stabilized. These findings suggest that the transcriptional activity of Ty3 retrotransposons may be closely linked to genome expansion. This result further supports the pivotal role of Ty3 retrotransposons in genome size evolution. However, the role of the Ty1 retrotransposon in genomes is rarely mentioned. In our analysis, we discovered that despite the high proportion of Ty3 retrotransposon elements and low proportion of Ty1 retrotransposon elements, components with transcriptionally active RT structures are predominantly Ty1 retrotransposon, accounting for approximately 50% of the total (Table 2). Additionally, Ty1 retrotransposon elements are primarily distributed in gene-rich regions (Figure 1 Track D, Figure 5c,d). Therefore, we hypothesize that the Ty1 retrotransposon superfamily may play a regulatory role for specific genes in the genome, maintaining high stability and transcriptional activity throughout evolution. In contrast, Ty3 retrotransposon elements show relatively unstable structure and function and are more prone to losing their transcriptional activity. These observations suggest distinct evolutionary dynamics and functional roles for Ty1 and Ty3 retrotransposons in the pepper genome.

In conclusion, we revealed key factors in genome size evolution through comparative analysis of two *Capsicum* genomes. WGD events did not cause genome size differences; instead, transposable element (TE) accumulation played the major role. Massive expansion of Ty3 retrotransposon elements represents the key driving force for genome expansion, while Ty1 retrotransposon elements, though present in lower proportions, maintain higher structural integrity and transcriptional activity and are primarily distributed in gene-rich regions, potentially playing important roles in gene regulation. The different evolutionary pathways of the Ty3 retrotransposon and the Ty1 retrotransposon elements collectively shape the structure and function of pepper genomes.

## Figures and Tables

**Table 1:** Comparative genomic statistics of two *Capsicum* species.

	Genome Size (Gb)	Chromosome Number	Gene Number	Total Gene Length (bp)
*Capsicum rhomboideum*	1.7	13	33,512	181,798,887
*Capsicum annuum cv. CM334*	2.9	12	31,600	142,495,079

**Table 2:** Comparison of transcriptionally active TEs and intact LTR-RT components between CrT2T and CM334 genomes.

	Transcriptionally Active TEs					Transcriptionally Active Intact LTR-RTs	
**Genome**	**Ty1**	**Ty3**	**Pararetrovirus**	**LINE**		**Ty1**	**Ty3**
CrT2T	981	730	89	0		177	121
CM334	687	358	44	182		73	24

## Data Availability

The original genome sequences used in this study are publicly available at the NCBI database. The *C. rhomboideum* genome can be accessed under the accession number GCA_031232105.1, and the *C. annuum* cv.CM334 genome is available under the accession number GCA_000512255.2. The transcriptome data used in this study can be accessed at SRA database.

## Supplementary Materials

The following supporting information can be downloaded at https://www.mdpi.com/article/10.3390/plants14142136/s1, Figure S1. Self-Synteny Dot Plots of CM334 and CrT2T. Figure S1A,B show the self-synteny dot plots of CM334 and CrT2T, respectively. Figure S2. KS frequency distribution of whole-genome duplication events in CM334 and CrT2T. Figure S2A,B show the self-synteny dot plots of CM334 and CrT2T, respectively. Figure S3. Dot Plot of Intergenomic Synteny between CrT2T and CM334. Figure S4. Chromosome-Level Synteny Map between CrT2T and CM334. Table S1. Non-redundant protein sequences used in the phylogenetic tree.

## Abbreviations

The following abbreviations are used in this manuscript:

TE	Transposable element
LTR-RT	Long terminal repeat retrotransposon
LTR	Long terminal repeat
RT	reverse transcriptase
WGD	Whole genome duplication
CrT2T	*Capsicum rhomboideum*
CM334	*Capsicum annuum* cv. CM334
