# Supplementary material for: The Dynamics of Long Terminal Repeat Retrotransposon Proliferation and Decay Drive the Evolution of Genome Size Variation in Capsicum
Source: Plants (Basel). 2025 Jul 10;14(14):2136. doi: 10.3390/plants14142136 (PMC12298681; doi:10.3390/plants14142136)
Supplement: Supplementary file 1 [file plants-14-02136-s001.zip › plants-3634664-supplementary.pdf]

Supplementary Material

1 Supplementary Figures and Tables

1.1 Supplementary Figures

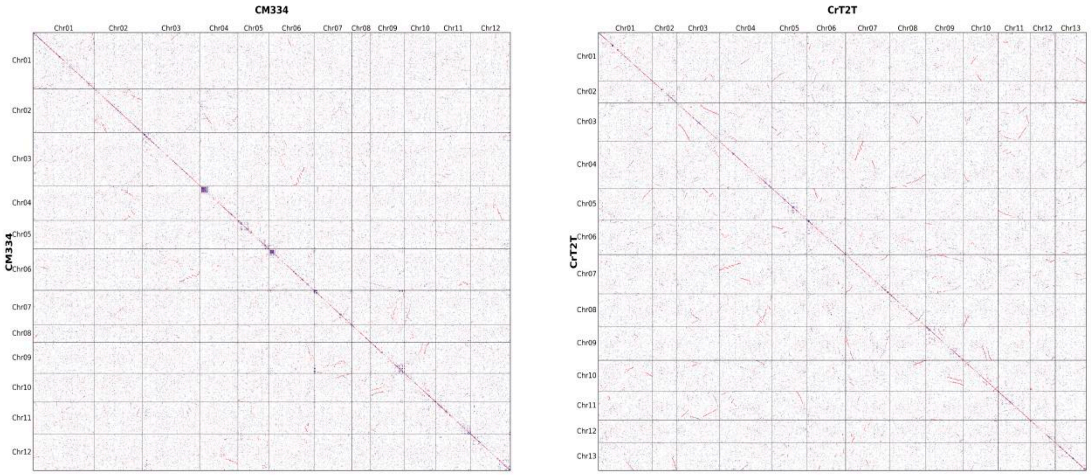

A B

Figure S1. Self-Synteny Dot Plots of CM334 and CrT2T. Figures S1A and S1B show the self-synteny dot plots of CM334 and CrT2T, respectively.

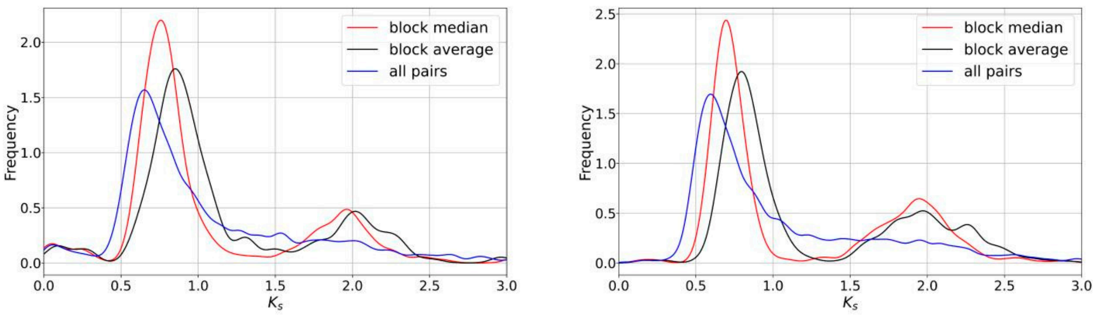

A B

Figure S2. KS frequency distribution of whole-genome duplication events in CM334 and CrT2T.Figures S2A and S2B show the self-synteny dot plots of CM334 and CrT2T, respectively.

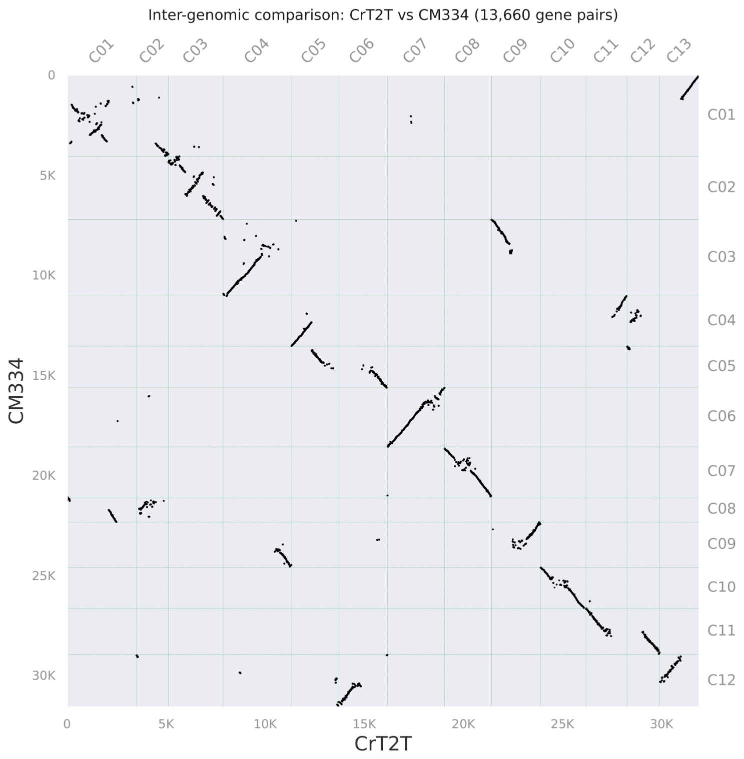

Figure S3. Dot Plot of Intergenomic Synteny between CrT2T and CM334

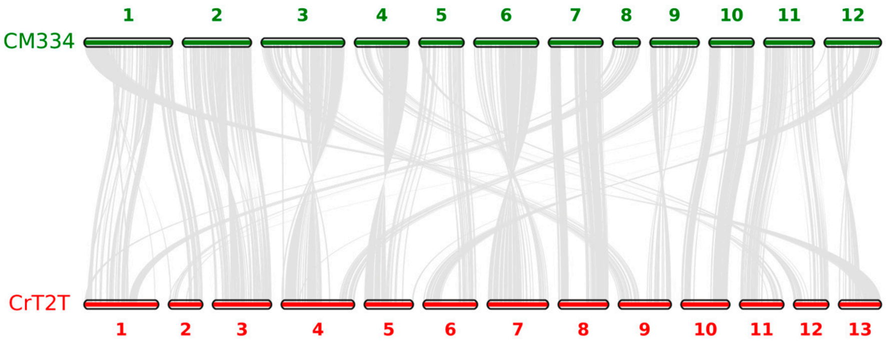

Figure S4. Chromosome-Level Synteny Map between CrT2T and CM334

1.2 Supplementary Table

Table S1. Non-redundant protein sequences used in the phylogenetic tree

	Genome Size	Accession number	Chromosome number	Ploidy Level	Database
C. annuum CM334	2.9G	GCA_000512255.2	12	diploid	https://www.ncbi.nlm.nih.gov/
C. annuum Ca59	3.0G	GCA_021292125.1	12	diploid	https://www.ncbi.nlm.nih.gov/
C.chinense PI159236	3.0G	GCA_002271895.2	12	diploid	https://www.ncbi.nlm.nih.gov/
C .baccatum	3.1G	GCA_002271885.2	12	diploid	https://www.ncbi.nlm.nih.gov/
C.pubescens Grif1614	3.7G		12	diploid	http://ted.bti.cornell.edu/pepper/
C.rhomboideum	1.7G		13	diploid	http://www.pepperbase.site/node/3
Physalis pubescens	1.4G	GWHANUX00000000	12	diploid	https://ngdc.cncb.ac.cn/gwh/
Solanum stenotomum F172	820Mb	GCF_019186545.1	12	diploid	https://www.ncbi.nlm.nih.gov/
Solanum verrucosum FXZP01	640Mb	GCF_900185275.1	12	diploid	https://www.ncbi.nlm.nih.gov/
Solanum tuberosum	684Mb	GCF_000226075.1	12	tetraploid	https://www.ncbi.nlm.nih.gov/
Solanum pinnatisectum LQN	642Mb	GCA_035578085.1	12	diploid	https://www.ncbi.nlm.nih.gov/
Solanum lycopersicum	800Mb	GCF_000188115.5	12	diploid	https://www.ncbi.nlm.nih.gov/
Solanum pennellii	895Mb	GCF_001406875.1	12	diploid	https://www.ncbi.nlm.nih.gov/
Nicotiana sylvestris	2.2G	GCF_000393655.1	12	diploid	https://www.ncbi.nlm.nih.gov/
Nicotiana tabacum TN90	3.5G	GCF_000715135.1	24	tetraploid	https://www.ncbi.nlm.nih.gov/
Nicotiana attenuata UT	2.3G	GCF_001879085.1	12	diploid	https://www.ncbi.nlm.nih.gov/
Nicotiana tomentosiformis	1.7G	GCF_000390325.2	12	diploid	https://www.ncbi.nlm.nih.gov/
Petunia axillaris	1.2G		7	diploid	https://solgenomics.net/
Ipomoea nil	711Mb	GCF_001879475.1	15	diploid	https://www.ncbi.nlm.nih.gov/
Coffea canephora	551Mb	GCA_900059795.1	9	diploid	https://www.ncbi.nlm.nih.gov/
Camellia sinensis	2.9G	GWHASIV00000000	15	diploid	https://ngdc.cncb.ac.cn/gwh/
Vitis vinifera	479Mb	GCF_030704535.1	19	diploid	https://www.ncbi.nlm.nih.gov/
Arabidopsis thaliana	116Mb	GCF_000001735.4	5	diploid	https://www.ncbi.nlm.nih.gov/
Oryza sativa	374Mb	GCF_034140825.1	12	diploid	https://www.ncbi.nlm.nih.gov/
